# Jasmonic acid ameliorates alkaline stress by improving growth performance, ascorbate glutathione cycle and glyoxylase system in maize seedlings

**DOI:** 10.1038/s41598-018-21097-3

**Published:** 2018-02-12

**Authors:** Mudaser Ahmad Mir, Riffat John, Mohammed Nasser Alyemeni, Pravej Alam, Parvaiz Ahmad

**Affiliations:** 1 Department of Botany, University of Kashmir, Srinagar, India; 2 Botany and Microbiology Department, College of Science, King Saud University, P. O. Box. 2460, Riyadh, 11451 Saudi Arabia; 3 Biology Department, College of Science and Humanities, Prince Sattam bin Abdulaziz University, 11942 Alkharj, Saudi Arabia; 4 Department of Botany, S.P. College, Srinagar, 190001 Jammu and Kashmir India

## Abstract

Environmental pollution by alkaline salts, such as Na_2_CO_3_, is a permanent problem in agriculture. Here, we examined the putative role of jasmonic acid (JA) in improving Na_2_CO_3_-stress tolerance in maize seedlings. Pretreatment of maize seedlings with JA was found to significantly mitigate the toxic effects of excessive Na_2_CO_3_ on photosynthesis- and plant growth-related parameters. The JA-induced improved tolerance could be attributed to decreased Na uptake and Na_2_CO_3_-induced oxidative damage by lowering the accumulation of reactive oxygen species and malondialdehyde. JA counteracted the salt-induced increase in proline and glutathione content, and significantly improved ascorbic acid content and redox status. The major antioxidant enzyme activities were largely stimulated by JA pretreatment in maize plants exposed to excessive alkaline salts. Additionally, increased activities of glyoxalases I and II were correlated with reduced levels of methylglyoxal in JA-pretreated alkaline-stressed maize plants. These results indicated that modifying the endogenous Na^+^ and K^+^ contents by JA pretreatment improved alkaline tolerance in maize plants by inhibiting Na uptake and regulating the antioxidant and glyoxalase systems, thereby demonstrating the important role of JA in mitigating heavy metal toxicity. Our findings may be useful in the development of alkali stress tolerant crops by genetic engineering of JA biosynthesis.

## Introduction

Agricultural soil contamination by alkaline salts has been recognized for the past few decades; however, exposure to alkalinity still continues and is worsening, principally in Asian countries^1^. In this context, alkaline stress is among the most crucial environmental constraints in arid and semi-arid environments, affecting agricultural crop productivity globally^2^. Excessive alkaline stress can induce numerous negative effects in plants at the cellular level by accumulating high Na and promoting ionic stress, osmotic stress by inducing water deficit, and ultimately resulting in the overproduction of reactive oxygen species (ROS) and oxidative stress^3–5^. Higher levels of alkaline salts in plant growth medium reduce K^+^ content and enhance Na uptake and accumulation, causing an efflux of K^+^ ions and promoting K^+^ leakage from plant cells^6–8^. Moreover, under alkaline stress conditions, Na content rises above that of K^+^, resulting in poor nutrient uptake and lack of Na/K homeostasis^8^. A slightly higher pH than the ideal level of alkaline salts is toxic and can adversely affect physio-biochemical processes, including mineral uptake, photosynthesis, membrane integrity, and yield of plants^3,4^. Higher pH also causes abnormality in root morphology, causing leaf chlorosis and necrotic lesions in leaves, all of which hamper plant growth and development, and ultimately lead to reduced crop yield^9^.

The inborn redox nature of alkaline salts, such as Na_2_CO_3_, also accelerates toxicity by generating ROS, such as superoxide anions (O_2_^·−^), hydrogen peroxide (H_2_O_2_), and hydroxyl radicals (OH^•^)^9,10^. To overcome alkaline-induced oxidative stress, plant cells are well furnished with inherent antioxidant capability that is comprised of enzymatic components, such as superoxide dismutase (SOD), glutathione peroxidase (GPX), catalase (CAT), ascorbate peroxidase (APX), glutathione *S-*transferase (GST), dehydroascorbate reductase (DHAR), monodehydroascorbate reductase (MDHAR), and glutathione reductase (GR), as well as non-enzymatic components, such as glutathione (GSH) and ascorbic acid (AsA)^11,12^. Being a highly reactive compound, Methylglyoxal (MG) accumulation is well known to occur under various abiotic stresses, including alkaline-salt stress^13^. Moreover, salt stress increases its cytotoxicity on biomolecules, and causes brutal oxidative damage to proteins either (i) directly through accelerating ROS production, or (ii) indirectly by the overproduction of advanced glycation end products (AGEs)^14,15^. To overcome MG toxicity, plants normally contain a GSH-dependent glyoxalase (Gly) system that converts MG into D-lactate by employing Gly I and Gly II^14^. Effective performance of antioxidant defence and the Gly systems corroborates enhanced tolerance to various abiotic stresses^13^. Maintaining Na and K homeostasis is critical in regulating the intracellular Na content to prevent toxicity. Plants normally have numerous mechanisms that hamper alkaline toxicity, such as inhibition of Na uptake, and prevention of its accumulation by binding with exudates of roots, intracellular sequestration by phytochelatins and strong ligands, such as cysteine-rich compounds^8^.

Maize (*Zea mays* L.) is an important cereal food crop grown worldwide and is also considered a high fibre yielding crop in many Asian countries, including China, India and Pakistan. India is the world’s largest producer of maize, with 8.4 million hectares under cultivation^16^. Cultivation of cereal crops, such as maize, in alkaline salt polluted lands, results in reduced yield and seed quality. In India, agricultural lands are exceedingly polluted with alkaline salts because of unrestrained water shortages and climate change^17^. Therefore, the development of maize varieties tolerant to alkaline stress or the elucidation of the entire mechanism of maize plant responses to alkaline toxicity is essential for sustainable maize production.

Plant responses to environmental stimuli are mostly orchestrated by an array of plant growth regulators, including phytohormones. Jasmonic acid (JA) and methyl jasmonate (MeJA) have been known to activate a number of signalling events during plant responses to abiotic and biotic stresses, thus developing improved safeguards in plants under these stresses^18^. Modification of endogenous JA levels in plants appeared to be a promising method of providing protection against numerous abiotic stresses, including salinity, heat, drought, and metal toxicity^18,19^. However, knowledge of the interactions of JA during alkaline stress and how JA modulates the physiological and biochemical changes under alkaline stress in an economically important cereal crop, such as maize, remains elusive. Moreover, available information regarding the precise roles of JA in its simultaneous regulation of ROS and detoxification of MG under alkaline stress in crop plants is limited, and thus warrants in-depth investigation to understand how antioxidant metabolism changes in crop plants in response to Na_2_CO_3_ induced-toxicity. In the current study, our goal was to investigate the effects of JA on growth and physio-biochemical processes in maize by considering the mechanisms related to (i) Na^+^ and K^+^ uptake and homeostasis, (ii) JA-induced alterations in growth performance and oxidative parameters, (iii) the role of JA in the modification of non-enzymatic and enzymatic defences, and (iv) MG detoxification under alkaline-stress conditions. The current study was undertaken to evaluate the effects of JA on growth, ion homeostasis, antioxidants, and methylglyoxal metabolizing enzyme activity in the amelioration of alkaline stress in maize plants.

## Results

### JA improves maize plant phenotypes

**Figure 1:**
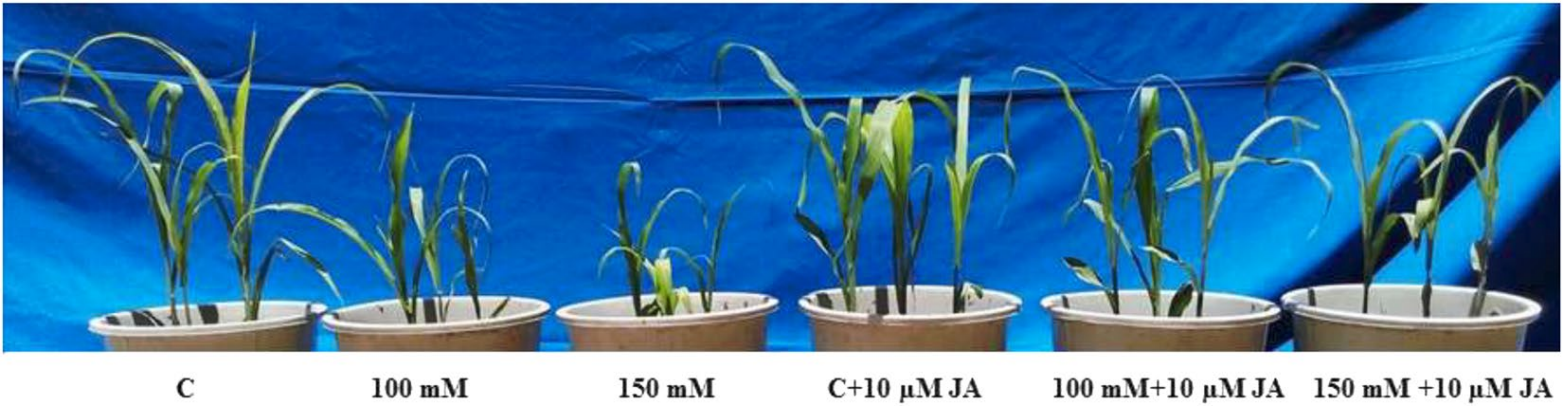
Effects of JA on phenotypes of maize plants in absence or presence of alkaline (Na_2_CO_3_) stress. C, 100 mM, 150 mM, C + JA, 100 mM + JA and 150 mM + JA correspond to control (0 mM), 100 mM Na_2_CO_3_, 150 mM Na_2_CO_3_, control + 10 µM JA, 100 mM Na_2_CO_3_ + 10 µM JA and 150 mM Na_2_CO_3_ + 10 µM JA respectively.

As depicted in Fig. 1, alkaline (Na_2_CO_3_) treatment induced yellowing symptoms on maize leaves. Alkaline treatment also hampered plant growth in terms of plant height and leaf length. Exogenous JA priming mitigated the negative effects of Na_2_CO_3_ and improved phenotypes of maize seedlings.

### Pretreatment with JA improves growth

**Figure 2:**
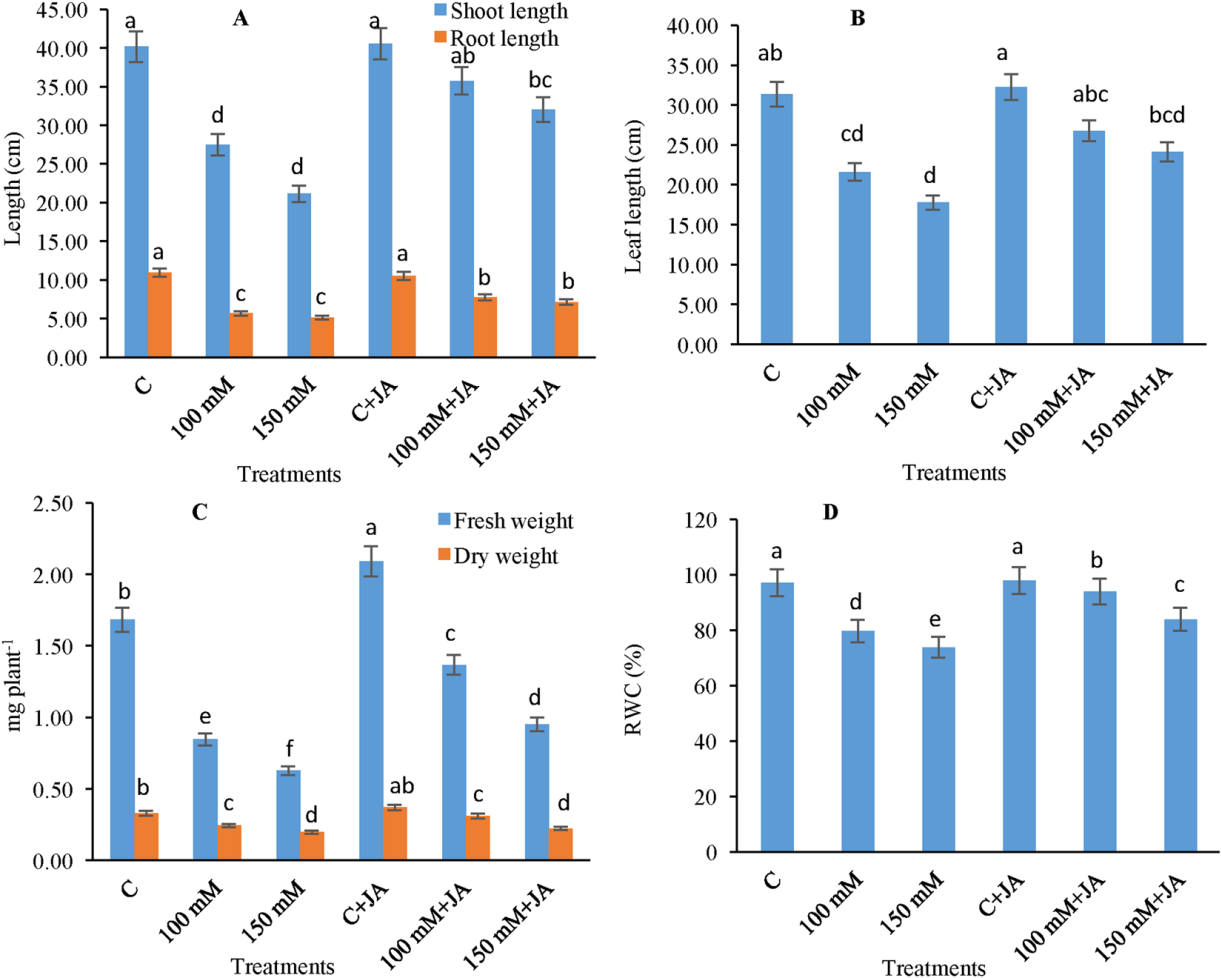
Effects of jasmonic acid (JA) on the (**A**) shoot and root length, (**B**) leaf length, (**C**) fresh and weight and (**D**) relative water content in maize plants with and without alkaline stress. Bars represent standard deviation (SD) of the mean (*n* = 3). Different letters (a, b, c, d, e and f) indicate statistically significant differences among the treatments, according to Duncan’s multiple range test at (*P* < 0.05). FW, fresh weight.

Seedlings fed only Na_2_CO_3_ showed reduced shoot height by 46.07 and 66.33% and reduced leaf length by 45.02 and 76.36% at 100 mM and 150 mM Na_2_CO_3_, respectively, as compared to that of the control (0 mM Na_2_CO_3_ + 0 µM JA). However, priming application of JA to alkaline treated plants relieved the toxic effects of Na_2_CO_3_ and enhanced the shoot height and leaf length of seedlings (Fig. 2A,B). Root length was reduced by 63.62 and 78.01% in alkaline treated plants in the 100 mM and 150 mM Na_2_CO_3_ treatments, respectively, in comparison with that of the untreated control (Fig. 2A). Pretreatment of JA enhanced root length by 16.91% at 100 mM Na_2_CO_3_ + 10 µM JA and 12.37% at 150 mM Na_2_CO_3_ + 10 µM JA relative to the 100 and 150 mM Na_2_CO_3_ treatments, respectively. Seedling biomass in terms of fresh and dry weights (FW and DW) indicated dramatic declines under alkaline stress (Fig. 2C). Seedling DW under Na_2_CO_3_ stress alone was decreased by 50.00% at 100 mM Na_2_CO_3,_ whereas at 150 mM Na_2_CO_3_ the maximum reduction in dry weight 90.90% was recorded (Fig. 2C). However, supplementation of JA to Na_2_CO_3_ fed seedlings resulted in improved DW, which demonstrated the positive effects of JA on alkaline stress.

### Seed priming with JA maintains RWC, chlorophyll pigments, soluble proteins, soluble sugars, and proline content

RWC under Na_2_CO_3_ stress significantly declined by 21.99% at 100 mM Na_2_CO_3_, but the maximum decline of 31.65% in RWC was recorded at 150 mM Na_2_CO_3_ compared with that of the untreated control (Fig. 2D). However, a notable increase in RWC (17.96 and 13.39%) was recorded for the 100 mM Na_2_CO_3_ + 10 µM JA and 150 mM Na_2_CO_3_ + 10 µM JA treatments, respectively, in the JA-primed alkaline-stressed seedlings compared with those fed only 100 mM Na_2_CO_3_ and 150 mM Na_2_CO_3_.

Data presented in Table 1, show that alkaline stress reduced the biosynthesis of photosynthetic pigments in maize leaves. The maximum reduction in total Chl and carotenoids of 67.79 and 60.00%, respectively, was recorded in the 150 mM Na_2_CO_3_ treatment as compared to that of the control untreated plants. Pretreatment with JA for alkaline stressed plants mitigated the toxic effects of Na_2_CO_3_ and improved the Chl content by 41.26 and 40.11% and carotenoids by 32.43 and 24.00% in 100 mM Na_2_CO_3_ + 10 µM JA and 150 mM Na_2_CO_3_ + 10 µM JA treatments, respectively, compared with that of 100 mM and 150 mM Na_2_CO_3_ treatments, respectively. Moreover, applying JA to unstressed 10 µM JA + 0 mM Na_2_CO_3_ plants resulted in enhanced Chl and carotenoid contents as compared to that of control untreated plants.

**Table 1:** Effects of jasmonic acid (JA) on the contents of total chlorophyll, carotenoids, water soluble proteins, soluble sugars and proline content in maize plants with or without alkaline stress. Values are means ± SD of three independent replications (*n* = 3). Different letters (a-e) within the column indicate statistically significant differences among the treatments, according to Duncan’s multiple range test at (*P* < 0.05). FW, fresh weight.

Treatment	Total chlorophyll (mg g^−1^ FW)	Carotenoids (mg g^−1^ FW)	Water soluble proteins (mg g^−1^ FW)	Soluble sugars (mg g^−1^ FW)	Proline (µmol g^−1^ FW)
Control	2.97 ± 0.13^a^	0.40 ± 0.02^b^	28.34 ± 1.68^a^	32.06 ± 1.83^e^	1.47 ± 0.18 ^cd^
100 mM	2.06 ± 0.12^b^	0.37 ± 0.03^bc^	25.09 ± 1.05^ab^	36.90 ± 1.76^c^	1.81 ± 0.14^b^
150 mM	1.77 ± 0.09^c^	0.25 ± 0.03^d^	21.99 ± 1.20^b^	40.82 ± 2.52^b^	3.73 ± 0.22^a^
C + JA	3.07 ± 0.14^a^	0.50 ± 0.03^a^	32.44 ± 1.62^a^	34.20 ± 1.75^d^	1.33 ± 0.16^d^
100 mM + JA	2.91 ± 0.11^a^	0.35 ± 0.04^bc^	25.86 ± 1.51^ab^	37.72 ± 1.51^c^	1.47 ± 0.18^d^
150 mM + JA	2.89 ± 0.17^a^	0.33 ± 0.03^c^	25.00 ± 0.94^ab^	46.02 ± 1.63^a^	1.61 ± 0.16^c^

The concentration of the osmoprotective proline significantly increased by 23.12% and 153.74% in 100 mM Na_2_CO_3_ and 150 mM Na_2_CO_3_ alkaline-stressed seedlings compared with that of the control (Table 1). Supplementation of JA to Na_2_CO_3_-treated seedlings showed a steep decline in proline content by 23.12% and 131.67%, respectively, at 100 mM Na_2_CO_3_ + 10 µM JA and 150 mM Na_2_CO_3_ + 10 µM JA as compared with only alkaline treated 100 and 150 mM Na_2_CO_3_ seedlings. However, proline content declined by 10.52% with supplementation of JA over the entire investigational period in non-stressed (10 µM JA + 0 mM Na_2_CO_3_) seedlings.

The Na_2_CO_3_ induced stress caused a significant reduction in total soluble sugars (TSSs) by 15.09 and 27.32% in 100 mM Na_2_CO_3_ and 150 mM Na_2_CO_3_ treatments, respectively, for maize seedlings. Application of JA mitigated the adverse effects of alkaline stress and caused significant TSSs accumulation of 2.22 and 12.73% in 100 mM Na_2_CO_3_ + 10 µM JA and 150 mM Na_2_CO_3_ + 10 µM JA treatments, respectively, compared to only alkaline stressed plants with 100 and 150 mM 10 µM JA + 0 mM Na_2_CO_3_, respectively (Table 1).

In the alkaline stressed plants, the soluble protein content was dramatically reduced by 17.64 and 28.87% in 100 and 150 mM Na_2_CO_3_ treatments, respectively, in comparison with that of 0 mM Na_2_CO_3_ + 0 µM JA untreated control plants. Applying JA to alkaline stressed plants alleviated the adverse effects of Na_2_CO_3_ and enhanced the total soluble protein content by 11.49 and 9.14% in the 10 µM JA + 100 mM Na_2_CO_3_ and 10 µM JA + 150 mM Na_2_CO_3_ treatments, respectively. Moreover, application of JA to unstressed maize plants increased protein content by 14.46% in 10 µM JA + 0 mM Na_2_CO_3_ treatment in comparison with that of the non-treated control (Table 1).

### JA attenuates Na^+^ toxicity, modulates Na^+^ and K^+^ homeostasis

**Figure 3:**
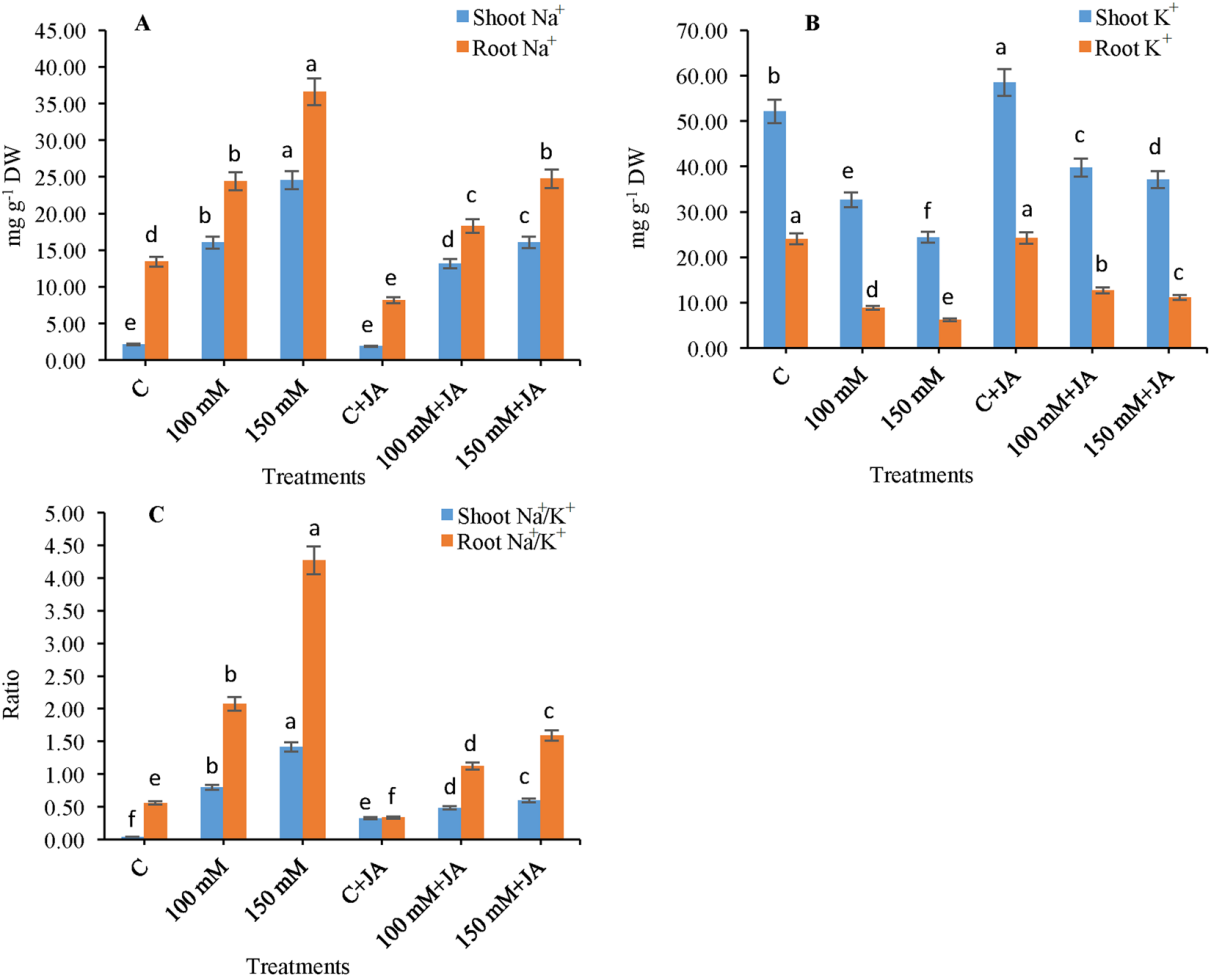
Effects of jasmonic acid (JA) on the (**A**) shoot and root Na^+^ content, (**B**) shoot and root K^+^ content, (**C**) shoot and root Na^+^/K^+^ ratio in maize plants with and without alkaline stress. Bars represent standard deviation (SD) of the mean (*n* = 3). Different letters (a, b, c, d, e and f) indicate statistically significant differences among the treatments, according to Duncan’s multiple range test at (*P* < 0.05). DW, dry weight.

In this study, exogenous JA caused a decline in Na^+^ uptake in roots, as well as in leaves of maize seedlings. The roots of only Na_2_CO_3_ fed seedlings exhibited enhancement in Na^+^ content by **6**.**5-fold** in 100 mM Na_2_CO_3_ and **10**.**5-fold** in 150 mM Na_2_CO_3_ treatments, as compared with that of the control (Fig. 3A). The uptake of Na^+^ declined by 21.88 and 52.80% in JA supplemented alkaline-fed 100 mM Na_2_CO_3_ + 10 µM JA and 150 mM Na_2_CO_3_ + 10 µM JA seedlings as compared to only 100 and 150 mM Na_2_CO_3_ treated seedlings, respectively (Fig. 3A).

Similarly, the leaves of 100 and 150 mM alkaline fed seedlings showed a **6**.**5-fold** and **10**.**5-fold** increase in Na^+^ content in comparison with that of the control group plants. However, pretreatment of JA to alkaline stressed seedlings resulted in a 21.94% and 52.69% reduction in Na^+^ content in 100 mM Na_2_CO_3_ + 10 µM JA and 150 mM Na_2_CO_3_ + 10 µM JA treatments, respectively, as compared to only 100 and 150 mM alkaline stressed seedlings (Fig. 3A). Furthermore, the JA primed seedlings showed decreased uptake of Na^+^ in leaves by 12.30% compared with that of the control seedlings.

Conversely, the reverse trend was noticed for K^+^ content in roots and the reduction in K^+^ content was **1**.**7** and **2**.**8-folds** in 100 and 150 mM Na_2_CO_3_, respectively, as compared to that of the untreated control plants (Fig. 3B). Supplementation with JA to 100 and 150 mM alkaline fed seedlings showed enhanced root K^+^ content by 43.51 and 78.49% as compared to only 100 and 150 mM Na_2_CO_3_ stressed plants. Likewise, a steep decline in shoot K^+^ content by 59.68 and 113.57% was recorded in the 100 and 150 mM alkaline fed maize plants. However, exogenous supplementation of JA to alkaline stressed seedlings exhibited a 21.94 and 52.07% increase in shoot K^+^ in 100 mM Na_2_CO_3_ + 10 µM JA and 150 mM Na_2_CO_3_ + 10 µM JA as compared to that of only 100 and 150 mM Na_2_CO_3_ fed plants (Fig. 3B). Additionally, the Na/K ratio in both roots and shoot was higher in the only 100 and 150 mM alkaline fed seedlings in comparison to that of the untreated control plants. Supplementation of JA to alkaline fed seedlings resulted in a significant reduction in the Na/K ratio in roots and leaves. The present data revealed that JA priming attenuates Na^+^ toxicity and protects seedlings from injuries (Fig. 3C).

### Effects of JA on H_2_O_2_ contents and malondialdehyde (MDA)

**Figure 4:**
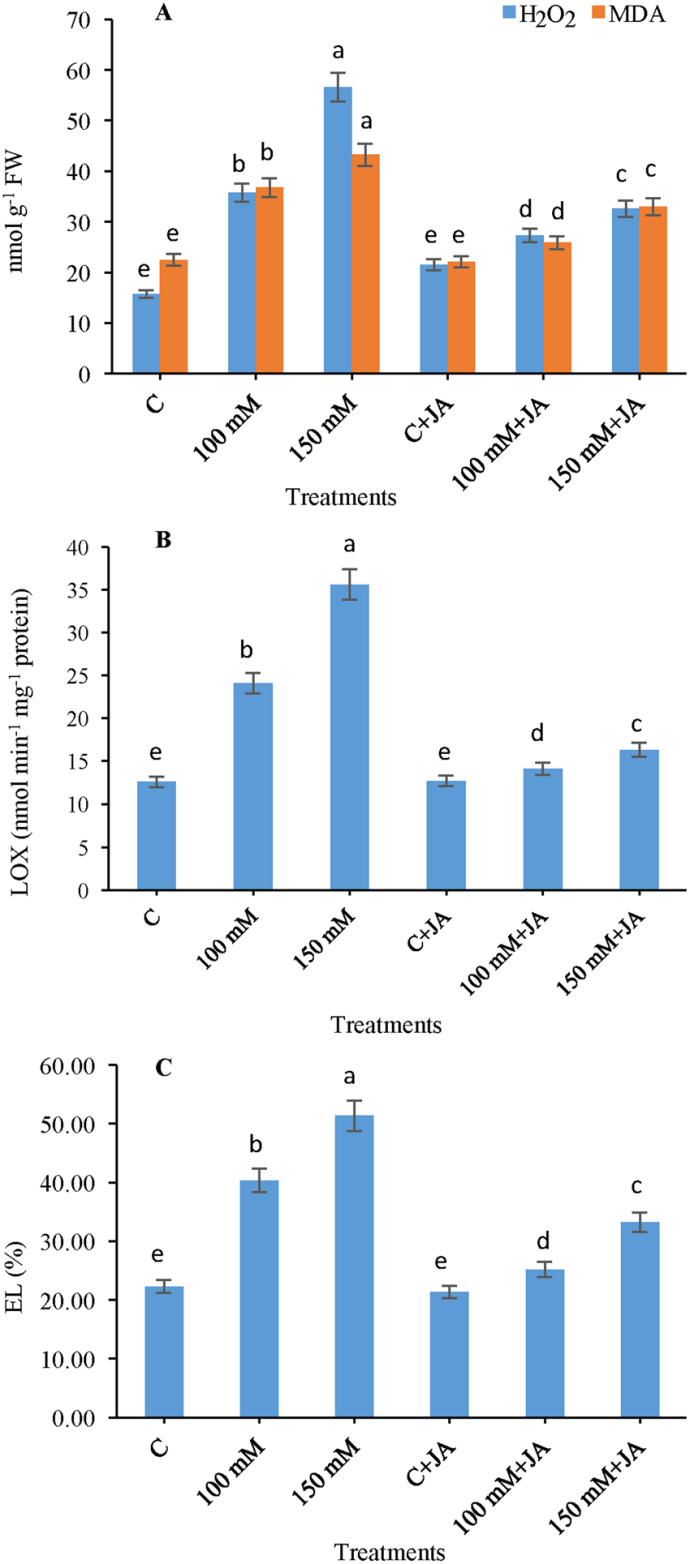
Effects of jasmonic acid (JA) on the (**A**) H_2_O_2_ and MDA content, (**B**) LOX and (**C**) EL in maize plants with and without alkaline stress. Bars represent standard deviation (SD) of the mean (*n* = 3). Different letters (a, b, c, d and e) indicate statistically significant differences among the treatments, according to Duncan’s multiple range test at (*P* < 0.05). FW, fresh weight.

The production of H_2_O_2_ was greatly increased by 96.85% with 100 Na_2_CO_3_, which furthermore was enhanced to 154.16% in the 150 mM Na_2_CO_3_ treatment as compared to that of the control untreated plants. Supplying JA to the Na_2_CO_3_ treated plants resulted in reductions in H_2_O_2_ production by 51.81 and 46.41% at 10 µM JA + 100 mM Na_2_CO_3_ and 10 µM JA + 150 mM Na_2_CO_3_, respectively, in comparison with only 100 and 150 mM Na_2_CO_3_ alkaline fed plants (Fig. 4A). In addition, under unstressed conditions, exogenous JA evinced a 0.69% increase in the contents of H_2_O_2_ for that of 10 µM JA + 0 mM Na_2_CO_3_, to ensure the protective signalling role of JA. Compared with untreated control seedlings, MDA content was increased by 63.45% in 100 mM Na_2_CO_3_ and 92.26% in 150 mM Na_2_CO_3_ stressed plants (Fig. 4A). However, application of JA to Na_2_CO_3_ fed plants showed a 17.29 and 21.36% reduction in lipid peroxidation at 100 mM Na_2_CO_3_ + 10 µM JA and 150 mM Na_2_CO_3_ + 10 µM JA, respectively, as compared to only alkaline-stressed plants at 100 and 150 mM Na_2_CO_3_, respectively.

### Effects of JA on lipoxygenase (LOX) activity and (%) electrolyte leakage (EL)

Lipoxygenase (LOX) activity was amplified by 99.44% in 100 mM Na_2_CO_3_ and 167.01% in 150 mM Na_2_CO_3_ fed maize plants, compared with that of the control (Fig. 4B). However, priming treatment of JA to alkaline fed plants showed a decrease in LOX activity by 77.94% at 10 µM JA + 100 mM Na_2_CO_3_ and 74.04% at 10 µM JA + 150 mM Na_2_CO_3_, compared with the 100 and 150 mM Na_2_CO_3_ treatments. In non-stressed conditions (10 µM JA + 0 mM Na_2_CO_3_), JA slightly altered LOX activity in seedlings relative to that of the control. The Na_2_CO_3_ treated plants exhibited an 81.10 and 130.47% increase in % EL as compared to that of the control seedlings at 100 and 150 mM Na_2_CO_3_, respectively, but pretreatment of JA to alkaline stressed plants showed a 60.18 and 54.52% reduction in EL at 100 mM Na_2_CO_3_ + 10 µM JA and 150 mM Na_2_CO_3_ + 10 µM JA, respectively, as compared to only Na_2_CO_3_ fed plants (Fig. 4C).

### JA maintains ascorbic acid (AsA) content, DHA, and AsA/DHA ratio

In comparison with that of the control (0 mM Na_2_CO_3_ + 0 µM JA) untreated plants, the level of total AsA declined significantly for both 100 and 150 mM Na_2_CO_3_-fed seedlings, with a steep decrease of 16.54 and 29.81% recorded for the 100 and 150 mM Na_2_CO_3_ treatments, respectively (Table 2). However, Na_2_CO_3_ stressed plants supplemented with JA showed enhancement in AsA concentrations by 9.94 and 3.54% more in 10 µM JA + 100 mM Na_2_CO_3_ and 10 µM JA + 150 mM Na_2_CO_3_ treatments, respectively, in comparison with alkaline only stressed plants 100 and 150 mM Na_2_CO_3_ respectively. The concentration of dehydroascorbic acid (DHA) significantly decreased by 40.55 and 35.11% in the 100 mM Na_2_CO_3_ and 150 mM Na_2_CO_3_ treatments, respectively, in alkaline stressed maize plants. However, JA pretreatment of Na_2_CO_3_ stressed plants resulted in the reduction in DHA concentration by 23.00 and 26.74% in the 100 mM Na_2_CO_3_ + 10 µM JA and 150 mM Na_2_CO_3_ + 10 µM JA treatments, respectively, as compared to alkaline only treated 100 and 150 mM Na_2_CO_3_ plants, respectively. The AsA/DHA ratio declined considerably in the alkaline-stressed seedlings at both 100 and 150 mM treatment levels, as compared to that of the control, and dropped severely by 63.86% at 100 mM Na_2_CO_3_ and 76.07% at 150 mM Na_2_CO_3;_ however, the ratios of AsA/DHA in the JA-primed alkaline-fed 10 µM JA + 100 mM Na_2_CO_3_ and 10 µM JA + 150 mM Na_2_CO_3_ seedlings was 35.40 and 31.37% higher compared with the only Na_2_CO_3_-treated seedlings at 100 and 150 mM Na_2_CO_3_ treatments, respectively, suggesting stress resistance (Table 2).

**Table 2:** Effects of jasmonic acid (JA) on the contents of non-enzymatic antioxidants, ascorbic acid (AsA), dehydroascorbate (DHA), AsA/DHA ratio, reduced glutathione (GSH), oxidized glutathione (GSSG) and GSH/GSSG ratio in maize plants with or without alkaline stress. Values are means ± SD of three independent replications (*n* = 3). Different letters (a–f) within the column indicate statistically significant differences among the treatments, according to Duncan’s multiple range test at (*P < 0*.05). FW, fresh weight.

Treatment	AsA (nmol g^−1^ FW)	DHA (nmol g^−1^ FW)	AsA/DHA ratio	GSH (nmol g^−1^ FW)	GSSG (nmol g^−1^ FW)	GSH/GSSG ratio
Control	210.52 ± 5.17^b^	46.90 ± 4.12^c^	4.49 ± 0.62^a^	159.25 ± 6.17^e^	12.50 ± 1.07^c^	12.82 ± 2.10^a^
100 mM	180.64 ± 6.22^d^	65.92 ± 6.08^a^	2.74 ± 0.42^d^	178.17 ± 6.22^c^	16.24 ± 2.07^b^	10.98 ± 1.19^b^
150 mM	162.07 ± 4.35 ^f^	54.37 ± 2.07^b^	2.98 ± 0.23^d^	168.74 ± 4.15^d^	19.34 ± 3.05^a^	8.73 ± 1.51^d^
C + JA	214.79 ± 6.34^a^	46.59 ± 3.10^c^	4.61 ± 0.51^a^	160.14 ± 3.34^e^	14.34 ± 1.04^bc^	11.18 ± 2.62^b^
100 mM + JA	198.60 ± 8.24^c^	53.59 ± 5.11^b^	3.71 ± 0.44^b^	198.38 ± 6.24^a^	19.01 ± 2.14^a^	10.49 ± 1.68^bc^
150 mM + JA	167.81 ± 5.78^e^	50.01 ± 4.12^c^	3.35 ± 0.21^c^	187.72 ± 4.65^b^	19.92 ± 2.45^a^	9.44 ± 3.78^d^

### Effects of JA on GSH content and GSH to oxidized GSH (GSSG) ratio (GSH/GSSG)

Maize seedlings stressed with Na_2_CO_3_ increased their GSH content by 11.88% with only 100 mM Na_2_CO_3_, compared with that of the control (Table 2). Applying JA to alkaline-stressed plants efficiently enhanced the GSH level up to 11.34 and 26.20% with 10 µM JA + 100 mM Na_2_CO_3_ and 10 µM JA + 150 mM Na_2_CO_3_, respectively, compared with only 100 and 150 mM alkaline-stressed plants. However, application of JA to non-stressed plants (10 µM JA + 0 mM Na_2_CO_3_) resulted in 13.12% higher levels of GSH contents as compared to that of the non-treated control. Alkaline stressed maize plants showed a dramatic decline in GSSG content by 29.92 and 54.72% with the 100 and 150 mM Na_2_CO_3_ treatments, respectively, when compared to that of the untreated control plants. Addition of JA to alkaline stressed plants resulted in 17.055 and 3.015% increase in GSSG concentration in comparison with that of only 100 and 150 mM Na_2_CO_3_ stressed plants, respectively. The alkaline only fed plants showed a 16.75 and 46.48% decrease in the GSH/GSSG ratio at 100 and 150 mM Na_2_CO_3_ treatment levels compared with that of the (0 mM Na_2_CO_3_ + 0 µM JA) control. However, application of JA to Na_2_CO_3_-stressed plants resulted in higher GSH/GSSG ratios than those of the only 100 and 150 mM Na_2_CO_3_-stressed seedlings at both 100 mM Na_2_CO_3_ + 10 µM JA and 150 mM Na_2_CO_3_ + 10 µM JA treatment levels (Table 2).

### Effects of JA on Antioxidant enzymes SOD, CAT, GPX, and GST

**Figure 5:**
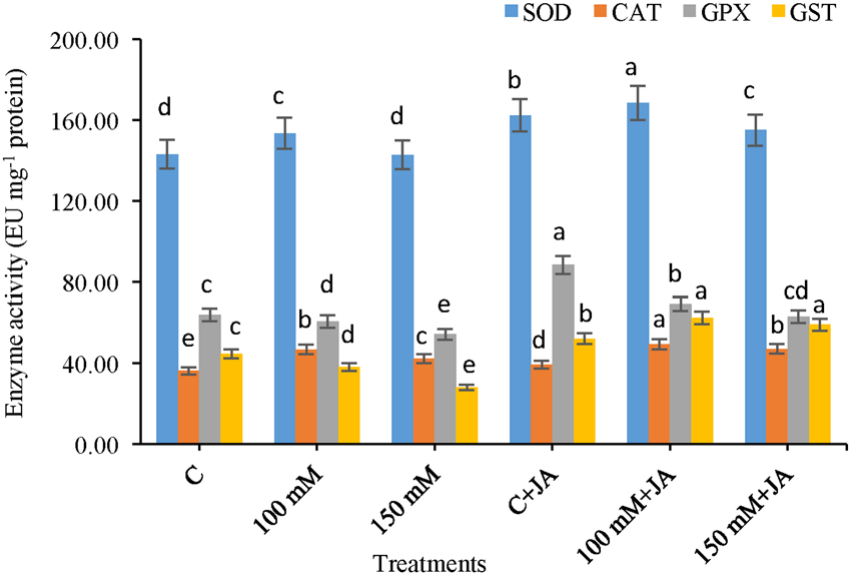
Effects of jasmonic acid (JA) on the activity of SOD, CAT, GPX and GST in maize plants with and without alkaline stress. Bars represent standard deviation (SD) of the mean (*n* = 3). Different letters (a, b, c, d and e) indicate statistically significant differences among the treatments, according to Duncan’s multiple range test at (*P* < 0.05).

The slight increase in superoxide dismutase activity 7.26% was recorded at 100 mM Na_2_CO_3_ level of treatment relative to that of the untreated control. JA-priming of alkaline stressed seedlings of maize was able to enhance SOD activity (9.82 and 8.59%) at both the 100 mM Na_2_CO_3_ + 10 µM JA and 150 mM Na_2_CO_3_ + 10 µM JA treatments, respectively, compared with only alkaline treatments of 100 mM and 150 mM, suggesting a complex effect of JA on modulation of SOD activity (Fig. 5).

The maize seedlings exposed to alkaline stress had increased CAT activity by 28.85 and 16.53% at 100 and 150 mM, respectively, as compared with that of the non-treated control (Fig. 5). In contrast, JA-supplementation to alkaline-stressed seedlings showed enhanced CAT activity by 5.40% at 100 mM Na_2_CO_3_ + 10 µM JA and 150 mM Na_2_CO_3_ + 10 µM JA, as compared to only 100 and 150 mM Na_2_CO_3_ stressed seedlings, respectively. Moreover, in comparison with that of the control (0 mM Na_2_CO_3_ + 0 µM JA) JA-priming to unstressed plants elevated catalase activity by 8.33% with the (0 mM Na_2_CO_3_ + 10 µM JA) treatment. The maize seedlings exposed to alkaline stress had reduced GPX activity by 5.26% at the 100 mM Na_2_CO_3_ and 17.56% at the 150 mM Na_2_CO_3_ level compared with control (Fig. 5). Exogenous JA to alkaline-fed plants had increased GPX activity by 14.06 and 15.85% at 100 mM Na_2_CO_3_ + 10 µM JA and 150 mM Na_2_CO_3_ + 10 µM JA levels compared with only seedlings treated with 100 and 150 mM Na_2_CO_3_ respectively. In non-stressed plants, application of JA enhanced GPX activity by 34.46% at (0 mM Na_2_CO_3_ + 10 µM JA) treatments in comparison with that that of the control plants. Seedlings exposed to Na_2_CO_3_ had decreased GST activity by 27.35 and 59.70% at 100 and 150 mM levels, respectively, compared with that of the control untreated plants (Fig. 5). In contrast, JA-priming to Na_2_CO_3_ stressed maize plants had 78.94 and 110.71% increased GST activity in 100 mM Na_2_CO_3_ + 10 µM JA and 150 mM Na_2_CO_3_ + 10 µM J treatments in comparison with only that of the 100 and 150 mM alkaline-stressed plants, respectively.

### Activities of ascorbate-glutathione cycle enzymes

**Figure 6:**
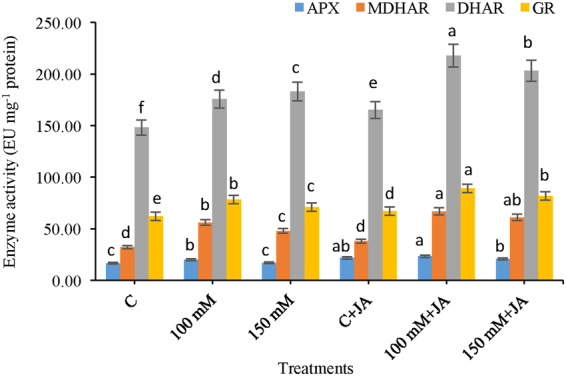
Effects of jasmonic acid (JA) on the activity of APX, MDHAR, DHAR and GR in maize plants with and without alkaline stress. Bars represent standard deviation (SD) of the mean (*n* = 3). Different letters (a, b, c, d, e and f) indicate statistically significant differences among the treatments, according to Duncan’s multiple range test at (*P* < 0.05).

The results related to the activities of the ascorbate-glutathione cycle enzymes are depicted in Fig. 6. Under alkaline stress, APX activity increased by 19.89 and 2.10% at 100 mM Na_2_CO_3_ + 10 µM JA and 150 mM Na_2_CO_3_ + 10 µM JA levels, respectively, compared with that of the control (Fig. 6). APX activity significantly increased by 17.29 and 21.36%, respectively, at 100 mM Na_2_CO_3_ + 10 µM JA and 150 mM Na_2_CO_3_ + 10 µM JA levels in the JA-pretreated alkaline-stressed seedlings compared with the 100 and 150 mM alkaline-stressed only seedlings, respectively. APX activity increased significantly in the JA-primed seedlings at (0 mM Na_2_CO_3_ + 10 µM JA) level only, relative to that of the control. MDHAR activity increased by 74.27 and 49.05% in 100 and 150 mM alkaline stressed seedlings compared with that of the control (Fig. 6). JA-priming of Na_2_CO_3_ stressed plants showed enhanced MDHAR activity by 19.34 and 27.33% in 100 mM Na_2_CO_3_ + 10 µM JA and 150 mM Na_2_CO_3_ + 10 µM JA treatments, respectively, in comparison with that of 100 and 150 mM Na_2_CO_3_ treatments. DHAR activity increased by 16.19 and 18.74% in 100 and 150 mM alkaline-stressed seedlings when compared with the control, respectively (Fig. 6). On the other hand, DHAR activity increased by 26 and 27% in the JA-pretreated Na_2_CO_3_ stressed seedlings compared with the alkaline-stressed only (100 mM Na_2_CO_3_) and (150 mM Na_2_CO_3_) treated seedlings, respectively. A significant change in DHAR activity was observed in the maize seedlings under non-stressed conditions upon JA pretreatment over the experimental period. GR activity decreased by 20.47 and 14.45% at 100 and 150 mM Na_2_CO_3_ stressed seedlings relative to that of the control respectively (Fig. 6). In addition, GR activity increased by 14.24 and 14.49% in the JA-pre-treated alkaline-stressed 100 mM Na_2_CO_3_ + 10 µM JA and 150 mM Na_2_CO_3_ + 10 µM JA seedlings compared with the 100 and 150 mM only alkaline fed seedlings.

### Modulation of glyoxalase system

**Figure 7:**
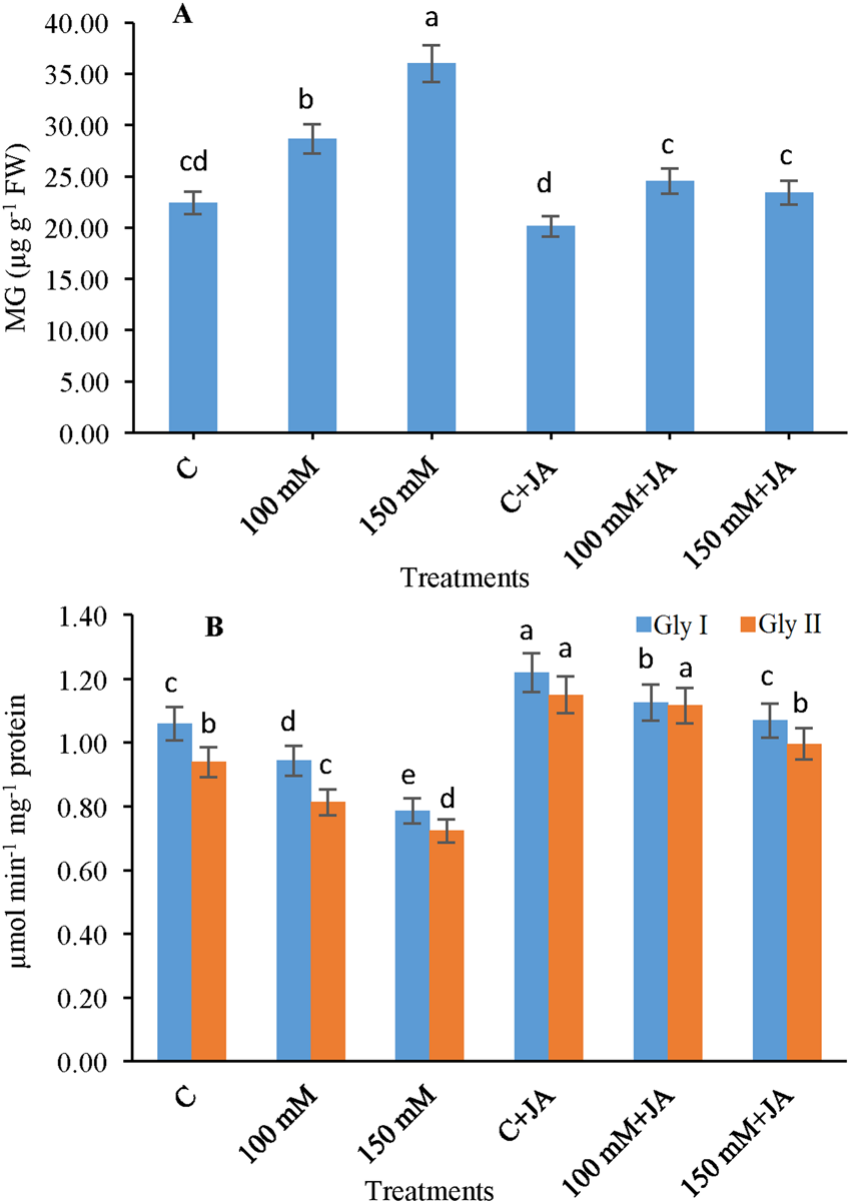
Effects of jasmonic acid (JA) on the (**A**) MG and (**B**) Gly I and Gly II in maize plants with and without alkaline stress. Bars represent standard deviation (SD) of the mean (*n* = 3). Different letters (a, b, c, d and e) indicate statistically significant differences among the treatments, according to Duncan’s multiple range test at (*P* < 0.05). FW, fresh weight.

Seedlings under alkaline stress linearly enhanced the concentration of MG by 27.75% at (100 mM Na_2_CO_3_) level and 56.71% at (150 mM Na_2_CO_3_) level compared with that of the control seedlings (Fig. 7A). However, application of JA to Na_2_CO_3_ stressed plants showed reduction in the levels of MG concentration by 32.48 and 27.98% at both (100 mM Na_2_CO_3_ + 10 µM JA) and (150 mM Na_2_CO_3_ + 10 µM JA) levels respectively, and declined the Na_2_CO_3_-induced toxicity. The activity of enzyme Gly I reduced by 12.74% at 100 mM and 34.17% at 150 mM alkaline stressed plants in comparison to control (0 mM Na_2_CO_3_ + 0 µM JA) plants (Fig. 7B). JA supplementation of alkaline stressed 100 mM Na_2_CO_3_ + 10 µM JA and 150 mM Na_2_CO_3_ + 10 µM JA treatments exhibited a 27.65 and 24.05% increase in Gly I activity relative to 100 and 150 mM Na_2_CO_3_ treatment levels, respectively, to ensure stress resistance. The activity of Gly II declined by 16.04% at 100 mM level and 30.55% at 150 mM level, in the Na_2_CO_3_-fed seedlings over that of the control (Fig. 7B). In addition, JA supplementation considerably amplified Gly II activity by 38.27 and 33.34% with 100 mM Na_2_CO_3_ + 10 µM JA and 150 mM Na_2_CO_3_ + 10 µM JA treatments, compared with the 100 and 150 mM Na_2_CO_3_- only fed seedlings, respectively. However, a significant rise in the activity of Gly II was evinced under non-stressed conditions upon JA-priming over control plants.

## Discussion

In this study, we have provided information regarding how JA regulates ion homeostasis to confer a shield on maize plants against high alkaline salts. Alkaline stress has been shown to hamper overall plant growth of maize plants (Fig. 1). In the current study, a dramatic decline was recorded in plant height, root length, leaf length, FW, and DW of maize seedlings fed with 150 mM alkaline salt, perhaps by accumulation of high Na content, caused osmotic stress by impaired ion homeostasis and hampered overall growth performance. However, pretreatment of JA to salt affected seedlings restored the plant growth and biomass. These results are in agreement with the findings of Keramat *et al*.^20,21^ in which exogenous JA counter abiotic stress constraints and restored growth. Alkaline stress dramatically reduced leaf relative water content (LRWC), and this might have been caused by osmotic stress, which stimulates the accumulation of osmoprotectant proline and induced physiological water deficit conditions and hampers water uptake. The priming treatment of both JA to alkaline stressed seedlings displayed improved RWC and maintains proline accumulation as high as the control group. This result is in consistent with the findings of Poonam *et al*.^22^ who reported that exogenous JA restored the RWC and Pro accumulation in *Cajanus cajan* copper-fed seedlings.

Alkalinity drastically declined the leaf chlorophyll pigments in the present study (Table 1). Our results were similar to those Abdel Latef and Tran^8,23^ who reported that alkaline toxicity reduced chlorophyll contents in *Morus alba* and Z. *mays* plants subjected to alkalinity. Reduction in chlorophyll pigments might been caused by inefficient activities of the enzymes proto chlorophyllide reductase and α-aminolevulinic acid dehydratase (ALA-dehydratase), which are coordinately involved in biosynthesis of chlorophyll^24^. Moreover, supplementation of JA improved shoot dry weight and chlorophyll content under multiple stress conditions^25^ in wheat^25^ and soybeans^21^. Accumulation of compatible osmolyte proline under alkaline stresses has been reported to be a noble indicator of stress tolerance^8,26^. Proline helps in osmotic adjustment, restoration of chlorophyll pigment molecules^27^. Application of JA is reported to enhance the proline content in *Glycine max* under Ni stress^21^. The enzymes related to cause accumulation of mRNAs encoding, proline-rich proteins might be stimulated by JA and protect the cell from the oxidative burst by scavenging ROS^28^.

H_2_O_2_ is a very noxious ROS and drastically increased with increasing Na_2_CO_3_ concentration and the results of the present study support the findings of Ahmad *et al*.^23^ in mulberry plants. Under salt stress, a product of membrane peroxidation MDA is frequently used as a prime indicator of oxidative stress^29^. Nahar *et al*.^13^ observed amplified MDA content because of a salt induced oxidative burst in mung beans. In addition, alkalinity was reported to increase H_2_O_2_ and LOX enzyme activity, which prompted lipid peroxidation. Furthermore, Abdel Latef and Tran^8^ also reported the enhancement in MDA content with higher alkaline concentrations in maize. Indeed, JA pre-treatment reduces the creation of H_2_O_2_ and other free radicals which openly affect the lipid membranes. The present result is supported by Sirhindi *et al*.^30^ who found overproduction in MDA content in soybean under Ni stress was significantly reduced by exogenous JA application. Poonam *et al*.^22^ have confirmed the reduced level of lipid peroxidation in *Cajanus cajan* to JA under copper toxicity. Therefore, pre-treatment of JA showed shielding nature on lipid membrane by decreasing production of H_2_O_2_ and superoxide radicles and alleviating lipid peroxidation by increasing the transcript levels and activities of SOD, POD, CAT and APX and the contents of GSH^25^.

The chief enzymatic network that detoxifies ROS is superoxide dismutase, catalase, peroxidases, and ascorbate-glutathione cycle enzymes^12^. The increase in antioxidants activities in the present study corroborates with the findings of Sirhindi *et al*.^21,22^. SOD is believed to serve as frontline antioxidant defence against various environmental stress regimes, including the salinity and catalysing O_2_^·−^ into H_2_O_2_, thereafter subsequently removed by CAT and GPX^12^. In present study, alkalinity-induced increased SOD activity with a negative correlation with O_2_ levels (Fig. 5), which indicates that potential SOD activity of this level might not have been efficient enough to neutralize the superoxide radicle. The current study demonstrated higher accumulation of H_2_O_2_ even after enhanced activities of the AsA-GSH cycle enzymes, proposing that accumulation of H_2_O_2_ exceeded ROS-scavenging potential in salt stressed maize plants (Figs 5 and 6). However, application of JA modulates the AsA-GSH glutathione cycle differentially, by maintaining APX, DHAR, and MDHAR activities above the untreated control level (Fig. 6). Furthermore, addition of JA and SA induced improvement in GR activity paid well maintained redox status possibly by regenerating GSH from GSSG, which supported with the observed increase in GSH level and GSG/GSSG ratio (Table 2). GSH dependent defence mechanisms are well known to play noteworthy roles in protecting plants from different environmental stresses, as well as salt stress^8,13^. Additionally, GSH neutralizes and detoxifies an extensive array of lipid peroxides and reactive aldehydes with the comfort of GST and GPX^31^. A vigilant analysis of ascorbate glutathione (GSH) allied defence mechanism under JA supplementation revealed that JA further increased of glutathione (GSH) level. This might have been because of the biosynthesis or up regulation of the activity of GR under salt stress, which thereafter participates in GPX and GST-mediated efficient detoxification of hydroperoxides (Figs 5 and 6). The present findings are in agreement with those of Rahman *et al*.^32^ and also reported the boosted activities of GPX and SOD in rice under salt stress might be caused by overproduction of free radicals (O_2_^·−^ and H_2_O_2_). However supplementation of JA stimulated mRNA levels of SOD and GPX and their activities under alkaline stress and reduced overproduction of O_2_^·−^ and H_2_O_2_ to reduce oxidative stress^21,25^.

Additionally, the present study revealed the effects of JA on GSH-dependent Gly system, which is involved in redox balance and particularly MG detoxification^33^. In present study, we observed that salt stress resulted in enhancement in MG concentration, which was possibly be caused by the incompetent and ineffective activities of Gly enzymes, as Gly I and Gly II activities, that decreased significantly as compared to that of the control (Fig. 7A,B). Moreover, an ineffective activity of Gly II might also entrap GSH thereby resulting in *S*-lactoylglutathione accumulation, which is highly cytotoxic^33^. However, exogenous application of JA exhibited efficient results on detoxification of MG, as evident by a correlation between reduced MG concentration and amplifying Gly enzyme activities, which thereafter protected cells from MG induced toxicity. Rahman *et al*.^32^ also reported the boosted activities of Gly enzymes (Gly I and II), and enzymatic antioxidants (APX, GPX, and GST) provided efficient salt tolerance in rice plants. Our results advocated that JA might improve maize plant tolerance to salt stress by harmonizing the biochemical activities of the enzymatic and non-enzymatic antioxidants and Gly systems to alleviate alkalinity-induced MG and ROS toxicity by decreasing the MG content and Na^+^ influx and K^+^ efflux through NSCC and GORK channels and increased nutrient uptake.

In conclusion, we found that supplementation of JA might be an efficient approach for successful tolerance of maize plants under alkaline stress based on the following motives. JA enhances photosynthetic potential by protecting pigments. Second, it maintains water balance and protects cells from oxidative bursting. Third, it diminishes oxidative injury by regulating antioxidant and GSH based Gly systems to detoxification of MG. Thus, our results establish a solid foundation that pretreatment of low dose JA in conveying agricultural land affected with salinity under cultivation will be a sustainable approach to enhance the crop yield.

## Methods

### Plant growth and treatments

Plants were grown under controlled conditions—temperature during day/night, 28/24 °C; light with photosynthetic photon-flux density, 200 µmol m^−2^ s^−1^; and humidity, 65% in a plant growth chamber. Seeds of *Z*. *mays* were surface sterilized with 0.1% mercuric chloride for 6 min and then rinsed five times with distilled water. The surface sterilized seeds were then primed with (i) distilled water and (ii) 10 µM JA for 12 h. JA was dissolved in 100% ethanol and then diluted to 10 µM for seed priming. Seed priming was done by soaking the seeds in 10 µM solution of JA and DW for control, for 12 hrs, thereafter air dried. The air-dried seeds of both groups were sown in plastic pots (five seeds/pot) filled with 3 kg of peat, perlite, and sand (1:1:1, v/v/v). After germination, pots were maintained with three seedlings per pot. The pots were arranged in a completely randomized design with three replications. Fifteen days after sowing, the JA primed and non-primed plants were exposed to 0 mM (control), 100 mM, and 150 mM Na_2_CO_3_ solution for an additional 10 days (25-day-old seedlings) in the specified conditions. Twenty-five days after sowing, maize plants were harvested to determine various physiological and biochemical responses.

The treatments used are given below:

- 0 mM (control)
- 100 mM Na_2_CO_3_
- 150 mM Na_2_CO_3_
- 0 mM (control) + JA
- 100 mM Na_2_CO_3_ + JA
- 150 mM Na_2_CO_3_ + JA

### Estimation of plant growth, biomass yield, and total chlorophyll

The root and shoot lengths were measured using a manual scale. For the measurement of DW seedlings were dried in oven at 70 °C for 48 h and then weighed. For the measurement of total chlorophyll of leaves, a 0.5 g leaf sample was homogenized in 5 mL acetone (80% v/v), followed by centrifugation at 10,000 × *g* for 8 min, after which the optical density was measured at 663 and 653 nm for Chl *a* and Chl *b*, respectively, using a spectrophotometer (Beckman 640 D, USA) by following the method of Arnon^34^.

### Analysis of LRWC and proline, total soluble protein, and soluble sugar contents

For analysing LRWC, fresh weight of leaves was measured after which the leaves were immediately placed between two pieces of filter paper and immersed in double distilled water for 24 h. After removing excess water by paper towel the turgid weight was measured by following the method of Barrs and Weatherley^35^.

To estimate the proline content the method of Bates *et al*.^36^ were employed. Freshly harvested leaf samples (0.5 g) were homogenized with 5 mL (3%) aqueous sulfosalicylic acid. The homogenate was then centrifuged at 11,500 × *g* for 12 min. The supernatant (1 mL) was thoroughly mixed with 1 mL glacial acetic acid and 1 mL acid ninhydrin. The reaction mixture was then boiled at 100 °C for 1 h and cooled to stop the reaction. The red colour that developed was removed with 2 mL toluene, and the optical density of the chromophore was measured at 520 nm using a spectrophotometer (Beckman 640 D, USA). Proline concentration was determined using a calibration curve of known proline concentrations.

For the estimation of total protein content, Lowry *et al*.^37^ were used and the optical density was recorded at 595 nm using a spectrophotometer (Beckman 640 D, USA) using bovine serum albumin as the control. For the estimation of TSSs, the absorbance was measured at 485 nm using a spectrophotometer (Beckman 640 D, USA) following the method of Dey^38^.

### Analysis of Na content and accumulation in the seedling roots and leaves

For the estimation of Na and K content, the separately harvested root and leaf samples were thoroughly washed with double distilled water to eliminate Na and K ions that might be adhering to the surface. The 0.1 g sample of oven dried (80 °C for 48 h) tissue was ground and digested with a HNO_3_:HClO_4_ (5:1 v/v) mixture at 80 °C until the yellow colour vanished. The content of Na and K in roots and leaves was analysed by flame atomic absorption spectrophotometry (Z-5000; Hitachi, Japan).

### Measurement of lipid peroxidation, H_2_O_2_ content, and % EL

Hydrogen peroxide (H_2_O_2_) was extracted and its content was determined after reaction with 0.1% TiCl_4_ in 20% H_2_SO_4_ Mostofa and Fujita^39^. For the measurement of lipid peroxidation the method of^40^ was followed. The production of MDA was measured, and the absorbance difference at 600 nm was detected using an extinction co-efficient of 155 mM^−1^ cm^−1^. Leaf EL was estimated by using the method of^41^ and the following formula:

1 $${\rm{EL}}( \% )=({{\rm{EC}}}_{1}/{{\rm{EC}}}_{2})\times 100$$

### Measurement of non-enzymatic antioxidants

Maize leaves (0.5 g) were homogenized in 3 mL ice-cold 5% meta-phosphoric acid containing 1 mM ethylenediaminetetraacetic acid (EDTA) using a mortar and pestle and centrifuged at 11,500 × *g* for 12 min at 4 °C. For the estimation of the total AsA concentration, the oxidized fraction was reduced by 0.1 M dithiothreitol. Reduced and total AsA levels were assayed at 265 nm in 100 mM K-phosphate buffer (pH 7.0) with 1.0 U ascorbate oxidase (AO) Dutilleul *et al*.^42^. DHA content was calculated by subtracting the reduced AsA amount from the total AsA content. The reduced GSH, GSSG, and total glutathione (GSH + GSSG) content were measured using the proposed method of Griffiths^43^. GSSG content was estimated after eliminating GSH by 2-vinylpyridine derivatization. GSH concentration was measured after deducting the value of GSSG from the total GSH content.

### Extraction and enzymes assays

Fresh maize leaves (1 g) were homogenized in 100 mM Tris-HCl in presence of 5 mM DTT (dithiothreitol), 10 mM MgCl_2_, 1 mM EDTA, 5 mM magnesium acetate, 1.5% polyvinylpyrrolidone (PVP-40), 1 mM phenylmethylsulfonyl fluoride, and 1 µg mL^−1^ aprotinin. The homogenate was centrifuged at 11,500 × *g* for 20 min at 4 °C and the resulting supernatants were used to estimate enzyme activities.

The activity of lipoxygenase (LOX, EC 1.13.11.12) was estimated by observing the increase in absorbance at 234 nm using linoleic acid as the substrate solution Doderer *et al*.^44^.

The activity of SOD (EC 1.15.1.1) was determined based on a xanthine-xanthine oxidase system El-Shabrawi *et al*.^45^. The reaction mixture contained 50 mM K-phosphate buffer, 2.24 mM nitroblue tetrazolium, 0.1 U CAT, 0.1 U xanthine oxidase, 2.36 mM xanthine, and enzyme extract. The activity of SOD was expressed as units of enzyme required to constrain 50% photoreduction of nitroblue tetrazolium min^−1^ mg^−1^ protein.

CAT (EC 1.11.1.6) activity was estimated according to the method of Aebi^46^ and the absorbance was measured at 240 nm. For the measurement of APX activity, the decrease in absorbance at 290 nm was monitored as AsA was oxidized Nakano and Asada^47^.

The MDHAR (EC 1.6.5.4) activity was estimated using 1 U AO and the oxidation rate of NADPH at 340 nm was measured Hossain *et al*.^48^.

The DHAR (EC 1.8.5.1) activity was determined by observing the formation of AsA from DHA at 265 nm using GSH Nakano and Asada^47^.

The activity of GR (EC 1.6.4.2) was measured by observing decreased absorbance at 340 nm for GSSG-dependent oxidation of NADPH by using the extinction coefficient of 6.2 mM^−1^ cm^−1^ ^49^.

The activity of GST (EC 2.5.1.18) was measured following the method of Hossain *et al*.^50^ by monitoring the increased absorbance at 340 nm.

The activity of GPX (EC: 1.11.1.9) was estimated by the method of Putter and Becker^51^ using H_2_O_2_ as the substrate.

The activity of Gly I (EC 4.4.1.5) was estimated according to the method of Hossain *et al*.^52^ with an extinction co-efficient of 3.37 mM^−1^ cm^−1^.

The activity of Gly II (EC 3.1.2.6) was estimated according to the method of Mostofa and Fujita *et al*.^39^ by employing an extinction co-efficient of 13.6 mM^−1^ cm^−1^.

### MG content estimation

For the estimation of MG content, the method described by Wild *et al*.^53^ was employed. Fresh maize leaves (0.5 g) were homogenized in 2.5 mL 0.5 M perchloric acid, incubated on ice for 15 min, and centrifuged at 11,200 × *g* at 4 °C for 10 min; thereafter, 1 mL of supernatant was transferred to a centrifuge tube. Charcoal (10 mg mL^−1^) was added and maintained at 24 °C for 15 min to decolourize. Then, the mixture was further centrifuged at 11,000 × *g* for 10 min, and saturated K_2_CO_3_ was added to the supernatant for neutralization. In a final volume of 1 mL, 650 μL neutralized supernatant, 330 μL of 100 mM sodium dihydrogen phosphate buffer (pH 7.0), and 20 μL freshly prepared 0.5 M N-acetyl-L-cysteine were added and incubated for 10 min, after which the optical density was recorded at 288 nm. MG content was calculated using a standard curve of known MG concentrations.

### Statistical analysis

Data were subjected to analysis of variance and Duncan’s multiple range test. The values represented are means ± SE (n = 3). Different letters indicate significant differences between treatments at *P* ≤ 0.05.
